# Genomic Characterization of the Japanese Indigenous Wine Grape *Vitis* sp. cv. Koshu

**DOI:** 10.3389/fpls.2020.532211

**Published:** 2020-11-05

**Authors:** Keisuke Tanaka, Yu Hamaguchi, Shunji Suzuki, Shinichi Enoki

**Affiliations:** ^1^NODAI Genome Research Center, Tokyo University of Agriculture, Tokyo, Japan; ^2^Laboratory of Fruit Genetic Engineering, The Institute of Enology and Viticulture, University of Yamanashi, Kofu, Japan

**Keywords:** *Vitis vinifera*, Koshu, wine grape, resequencing, single-nucleotide variation, insertion/deletion, copy number variation, presence/absence variation

## Abstract

*Vitis* sp. cv. Koshu is indigenous to Japan and used as a table and processing grape. It also constitutes an important grape cultivar in Japanese white wine making and is phylogenetically distinct from European grapes. To understand its genomic information, we compared its small and structural variations with those of the table grape cultivar “Thompson seedless” and European wine grape cultivar “Tannat” via a short-read-based resequencing approach. The Koshu genome exhibited high heterozygosity compared to these cultivars, with this characteristic being particularly prominent on chromosome 7. Furthermore, Koshu structural variation encompassed the most and largest extent of duplications and the fewest and smallest extent of deletions with regard to copy number variation and the fewest absence variations among the compared grape cultivars. Plant disease resistance related to cell death associated with hypersensitive response and environmental stress response, such as water deprivation, oxidative stress, and cell wall organization, was inferred through enrichment analysis of small and structural variations. Variant accumulation levels in Koshu indicated that phenylpropanoid, flavonoid, glutathione, and α-linolenic acid pathways were related to polyphenol and flavor components. Together, this Koshu genomic information provides a foundation for improving the quality of Koshu wine and may facilitate the use of Koshu as a genetic resource.

## Introduction

The berries from grapevine, an angiosperm and deciduous shrub, have substantively contributed to human food culture. Table grapes are eaten raw and used in processed food manufacturing, whereas wine grapes are primarily used in wine making. Wine grapes encompass a large number of cultivars with European wine grapes (*Vitis vinifera* L.) being composed of 12,759 varieties, of which 1,368 cultivars are used in wine making (Robinson et al., 2012)^1^. Among these, *V*. *vinifera* cv. Pinot Noir, which is indigenous to Burgundy, France, is one of the most popular grape cultivars because its berries are used to produce red wines that are widely appreciated by consumers worldwide. Accordingly, this cultivar has been studied in various research fields including basic biology, breeding science, viticulture, and enology. In particular, the *de novo* construction of the genomic sequence of its highly homozygous clone (PN40024) using a whole-genome shotgun strategy represented the first such effort in fruit trees and grape cultivars (Jaillon et al., 2007; Velasco et al., 2007). The “whole-genome resequencing” approach can exhaustively clarify variations such as single-nucleotide variation (SNV), insertion/deletion (InDel), copy number variation (CNV), and presence/absence variation (PAV) through comparison with an object organism genome as the reference genome. Thus, present-day grape research can be approached from the genomic level and trait-associated genetic differences between individuals or lineages can be predicted or determined from such polymorphic information.

*Vitis* sp. cv. Koshu, a hybrid of *Vitis vinifera* L. and *V*. *davidii* Foex, is used as a table grape and processing grape for juice, frozen treat, and jam production, for example. Notably, it also constitutes an important grape cultivar in Japanese white wine making. Recently, Koshu wines have received accolades in international wine competitions. Koshu is a Japanese indigenous cultivar that has been cultivated mainly in Yamanashi Prefecture, Japan (Supplementary Figure S1). It was derived from a European wine grape cultivar via the Silk Road and crossed with the Chinese wild species *V*. *davidii* before being introduced from the continent (Goto-Yamamoto et al., 2015). To improve Koshu grape and wine quality, researchers have not only investigated such historical and systematic backgrounds but also performed chemical and biological characterizations to uncover agronomic and enological traits. As for the morphological characteristics of Koshu berry, its skin is pink, and its size is two times that of Pinot Noir. Consistent with this, the transcriptional profiles of Koshu suggest that the ubiquitin-proteasome system induces skin cell expansion during ripening by degrading auxin/indole-3-acetic acid family proteins (Kobayashi et al., 2009). Conversely, Chi3K, a chitinase gene derived from Koshu, is highly similar to class III chitinase in Pinot Noir and inhibits the growth of *Botrytis cinerea*, the causative agent of gray mold disease in grapes (Ano et al., 2003). In turn, as the amounts of two phenolic compounds, hydroxycinnamic acid and monomeric flavonol, in Koshu at harvest were larger than those in European wine grape cultivars (Kobayashi et al., 2011), Koshu white wine has a higher total phenolic content than white wines made from European white cultivars and exhibits slight astringency. Furthermore, through comparative transcriptome analysis we revealed that the high expression of some genes involved in flavonoid biosynthesis and glutathione metabolism is related to wine quality (Enoki et al., 2018). Nevertheless, although such studies have elucidated several functional mechanisms, the whole genomic information of Koshu has remained unknown. Alternatively, a good understanding of the Koshu genome would be expected to lead to improvements in quality of the Koshu plant and berry for breeding, cultivation, and wine making.

In this study, we first compared whole chloroplast genomes to estimate the phylogenetic relationship between Koshu and other grape cultivars or clones. Whole-genome resequencing analysis was performed to understand the genomic variations of Koshu using PN40024 as the reference genome. In addition, table grape cultivar “Thompson Seedless,” also termed Sultanina, and European wine grape cultivar “Tannat,” which have existing sequence data, were utilized for detailed comparison with the Koshu genome because the Koshu grape is used for multiple purposes. Specifically, the Thompson Seedless is a white grape similar to Koshu and used for various purposes, whereas Tannat is used for winemaking as a black grape. The characteristics of Koshu that differed from those of the other grape cultivars were estimated using gene variants.

## Materials and Methods

### Plant Material and DNA Extraction

Fresh leaves of Koshu were collected at the Yamanashi Fruit Tree Research Station (Yamanashi, Japan). Approximately 90 g of fresh leaves was ground in liquid nitrogen, and the fine powder was transferred to a suspension buffer (10 mM Tris-HCl at pH 9.5, 80 mM KCl, 10 mM ethylenediaminetetraacetic acid, 10 mM spermidine, 0.5 M sucrose, 0.5% Triton X-100, and 0.15% 2-mercaptoethanol). After the powder was filtered through Miracloth (Merck Millipore), the filtrate was centrifuged at 2,000 × *g* for 10 min. The collected pellet was resuspended in the suspension buffer, and genomic DNA was extracted using the CTAB method (Doyle and Doyle, 1987). The extracted DNA was purified using a NucleoBond AXG 100 column (Macherey-Nagel, Düren, Germany). The purity and concentration of the extracted DNA were measured using a NanoDrop spectrophotometer (Thermo Fisher Scientific, Wilmington, DE, United States) and a Qubit fluorometer (Thermo Fisher Scientific) with a Quant-iT dsDNA BR Assay Kit (Thermo Fisher Scientific). Genomic DNA fragments were visualized by agarose gel electrophoresis and pulsed-field gel electrophoresis.

### Library Construction and Sequencing

For the library construction prior to paired-end sequencing, 1 μg of genomic DNA was fragmented by shearing to an average fragment size of 400 bp using an Adaptive Focused Acoustics sonicator (Covaris, Woburn, MA, United States). After purification, the paired-end DNA library was constructed using a TruSeq DNA Sample Preparation Kit v2 (Illumina, San Diego, CA, United States). The fragmented DNA was end-repaired, dA-tailed, and ligated with the paired-end adapter according to the manufacturer’s instructions. The adapter-ligated DNA was amplified by 10 cycles of high-fidelity polymerase chain reaction (PCR) amplification, and the amplified product was size-selected using agarose gel electrophoresis with the approximate insert size set to 300–400 bp. Library quality and concentration were assessed using an Agilent Bioanalyzer 2,100 (Agilent Technologies, Waldbronn, Germany) and an Agilent DNA 1,000 kit.

The paired-end library were sequenced by 200 cycles (2 × 100 bp) using the Genome Analyzer IIx system (Illumina). Reads in FASTQ format were generated using the CASAVA v1.8 pipeline (Illumina). The sequenced read data were submitted to the NCBI Sequence Read Archive database (SRA)^2^ with the accession number SRR11234330.

### Collection of SRA Entries Derived From Other Grape Cultivars and Clones

Accession numbers for a total of 127 *V*. *vinifera* entries were retrieved from the NCBI SRA database (Supplementary Table S1). Among these, genomic read data from the Thompson Seedless (accession numbers SRX316886 and SRX316887; Di Genova et al., 2014) and Tannat (accession numbers SRX283507 and SRX283818; Da Silva et al., 2013) cultivars were used for comparison of Koshu with European grape cultivars.

### Phylogenetic Analysis

Briefly, read data were processed using CLC Genomics Workbench 9.0 (Qiagen, Hilden, Germany). Read data from Koshu and 127 screened SRA entries were cleaned by trimming adapter sequences and quality filtering and then were mapped to the *V*. *vinifera* chloroplast genome (NC_007957.1) of the NCBI Reference Sequence Database (RefSeq)^3^. The mapping parameters were as follows: mismatch cost = 2, insertion cost = 3, deletion cost = 3, length fraction = 0.9, and similarity fraction = 0.9. After local realignment of the mapped reads, duplicate PCR reads were discarded. Subsequently, consensus sequences between the chloroplast genome of each screened SRA entry and that of Koshu were reconstructed using the default parameters. Multiple sequence alignment among the sequence data was computed using MAFFT v7.157b (Katoh and Standley, 2013). To infer phylogenetic relationships among the selected entries, a neighbor-joining tree was reconstructed via the nucleotide distance measure of the Kimura 80 model with 1,000 bootstrap replicates using CLC Genomics Workbench 9.0.

### Read Mapping to the Reference Whole Genome

The reads of Koshu, Thompson Seedless, and Tannat were mapped to the PN40024 reference genome; the whole-genome sequence (assembly accession number GCF_000003745.3) and annotation data (annotation release 102) of Pinot Noir were retrieved from the NCBI Genome database^4^. The mapping, local realignment, and PCR duplicate removal were carried out similar to the procedures described in section “Phylogenetic Analysis” using CLC Genomics Workbench 9.0.

### Small Variant Detection

Variant calling based on SNV and InDel was performed using the CLC Genomics Workbench built-in tool “Fixed Ploidy Variant Detection.” The calling parameters were as follows: ploidy = 2, required variant probability = 90.0, minimum coverage = 10, minimum frequency = 20, minimum central quality = 40, and minimum neighborhood quality = 30. Furthermore, high-quality variants were selected using QUAL = 80. Genes with non-synonymous substitutions in the selected variants were enriched as Gene Ontology (GO) and Kyoto Encyclopedia of Genes and Genomes (KEGG) ontology (KO) terms using the web-based tool “DAVID 6.8”^5^ (Huang et al., 2007). The enriched terms were statistically analyzed using the modified Fisher’s exact test (*p* < 0.05) contained in the tool. The ratio of non-synonymous to synonymous substitutions (dN/dS) was plotted using the web-based tool BoxPlotR^6^ (Spitzer et al., 2014). A heatmap was drawn using the R package “gplots” (Warnes et al., 2015) for GO and KO terms that met the significance level (*p* < 0.05) in any one of the three cultivars. The number of non-synonymous substitutions in each gene for the three cultivars was visualized using the R package “Pathview” (Luo and Brouwer, 2013).

### Structural Variant Detection

Variant calling based on CNV was performed using CNVnator version 0.3.3 (Abyzov et al., 2011). The calling parameters were as follows: size ≥4,000, normalized RD ≥2 or ≤0.5, and *E*-value by *t*-test statistics < 0.05. In comparison, variant calling based on PAV was run using the CLC Genomics Workbench built-in tool “InDel and Structural Variants” The genes contained in the identified regions were enriched as GO and KO terms using the web-based tool “DAVID 6.8” The enriched terms were statistically analyzed using the modified Fisher’s exact test (*p* < 0.05) contained in the tool.

## Results

### Phylogenetic Relationships Based on the Whole Chloroplast Genome

The phylogenetic relationships of Koshu and 127 screened SRA entries were estimated based on the whole chloroplast genome (Supplementary Figure S2). The dendrogram was divided mainly into three clades. Although Koshu was related to the clade including Pinot Noir, it was not completely united therewith and showed an independent divergence. In comparison, Thompson Seedless and Tannat, which were used in the comparative genome analysis, were related to different clades from that of Koshu.

### Small Variations Based on SNV and InDel

We utilized a high-throughput DNA sequencing approach to clarify the genomic information of Koshu. The sequenced data outputted approximately 96.63 Gb of total nucleotides of through the paired-end and mate pair sequencing approaches (Supplementary Table S2). We prepared the clean reads of the two European *V*. *vinifera* cultivars, Thompson Seedless and Tannat, for comparison with Koshu. The mapping efficiency of Koshu, Thompson Seedless, and Tannat to the PN40024 reference genome was 88.71, 91.60, and 93.39%, respectively. The average read depth of the cultivars was 79.80×, 73.36×, and 84.94×, respectively, and the cover rate was 94, 93, and 94%.

After local realignment and duplicate removal, 1,443,211 SNVs and 325,949 InDels for Koshu, 1,179,240 SNVs and 323,089 InDels for Thompson Seedless, and 2,127,929 SNVs and 343,826 InDels for Tannat were detected by filtering the required variant probability, minimum coverage, minimum variant frequency, base quality, relative read direction, and mapping quality score (Supplementary Table S3). The SNV substitutions showed almost the same pattern distribution among all tested cultivars, and the transition rate was nearly twofold that of the transversion (Supplementary Figure S3A). All SNV substitutions exhibited the highest frequencies for Tannat and the lowest frequencies for Thompson Seedless in proportion to the number of SNVs. The InDel size distribution indicated that deletions of a given size were more frequent than insertions for all the cultivars and that smaller nucleotide lengths were more likely to exhibit numerical variation (Supplementary Figure S3B). The 2-bp deletion was most common only in the deletion size distribution for Koshu whereas the 1-bp deletion was prevalent in other cultivars.

**FIGURE 1:**
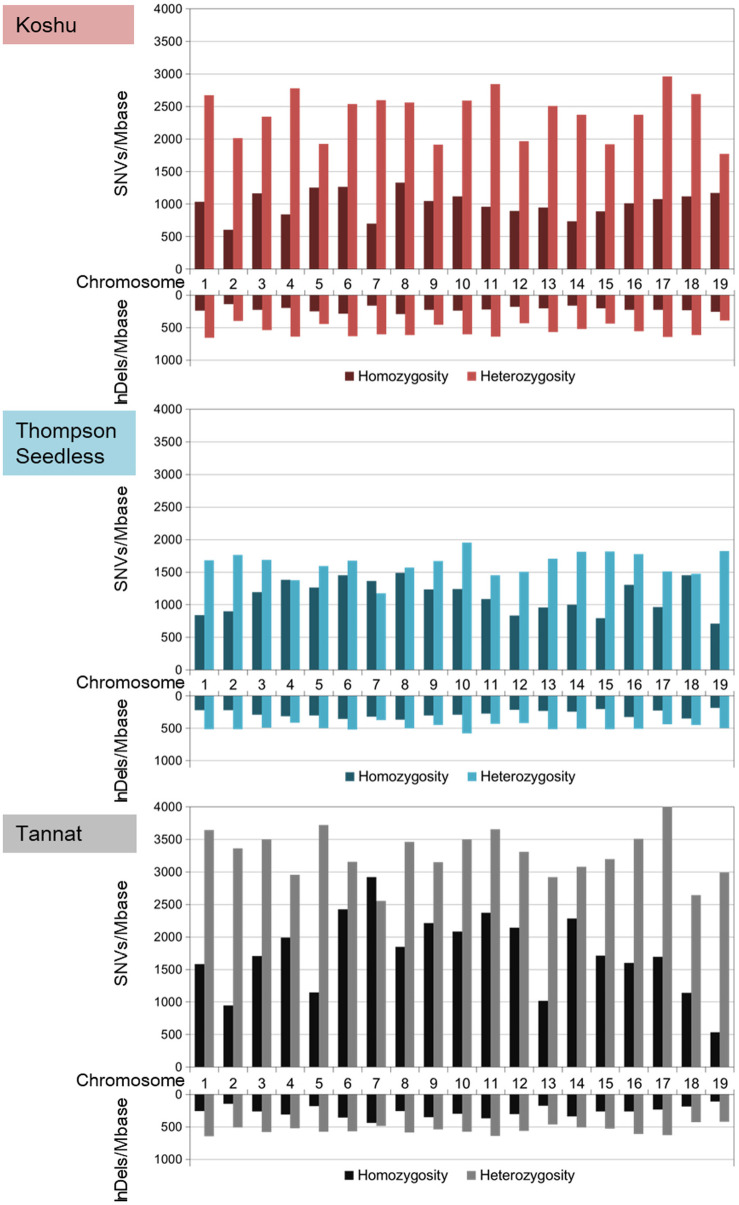
Frequencies per Mb of homozygous and heterozygous variants based on SNVs and InDels in each chromosome for Koshu, Thompson Seedless, and Tannat.

The ratios of heterozygous/homozygous variants were the highest for Koshu over the whole genome (Figure 1 and Supplementary Table S3). In chromosome 7, the heterozygous/homozygous ratios of the SNV variants for Thompson Seedless and Tannat were <1. In contrast, Koshu exhibited higher heterozygosity than homozygosity on all chromosomes. The average variant frequencies including SNVs and InDels were 4,159 variations/Mb in Koshu, 3,521 variations/Mb in Thompson Seedless, and 5,849 variations/Mb in Tannat. The highest SNV and InDel frequencies per Mb in Koshu, Thompson Seedless, and Tannat were found on Chr17 (4,035 SNVs/Mb) and Chr6 (916 InDels/Mb), Chr10 (3,189 SNVs/Mb) and Chr6 (878 InDels/Mb), and Chr11 (6,025 SNVs/Mb) and Chr11 (1,006 InDels/Mb), respectively. Conversely, the lowest SNV and InDel frequencies per Mb were found on Chr2 (2,613 SNVs/Mb and 540 InDels/Mb), Chr12 (2,341 SNVs/Mb and 633 InDels/Mb), and Chr19 (3,525 SNVs/Mb and 527 InDels/Mb), respectively.

### Functional Annotation of Small Variations

**FIGURE 2:**
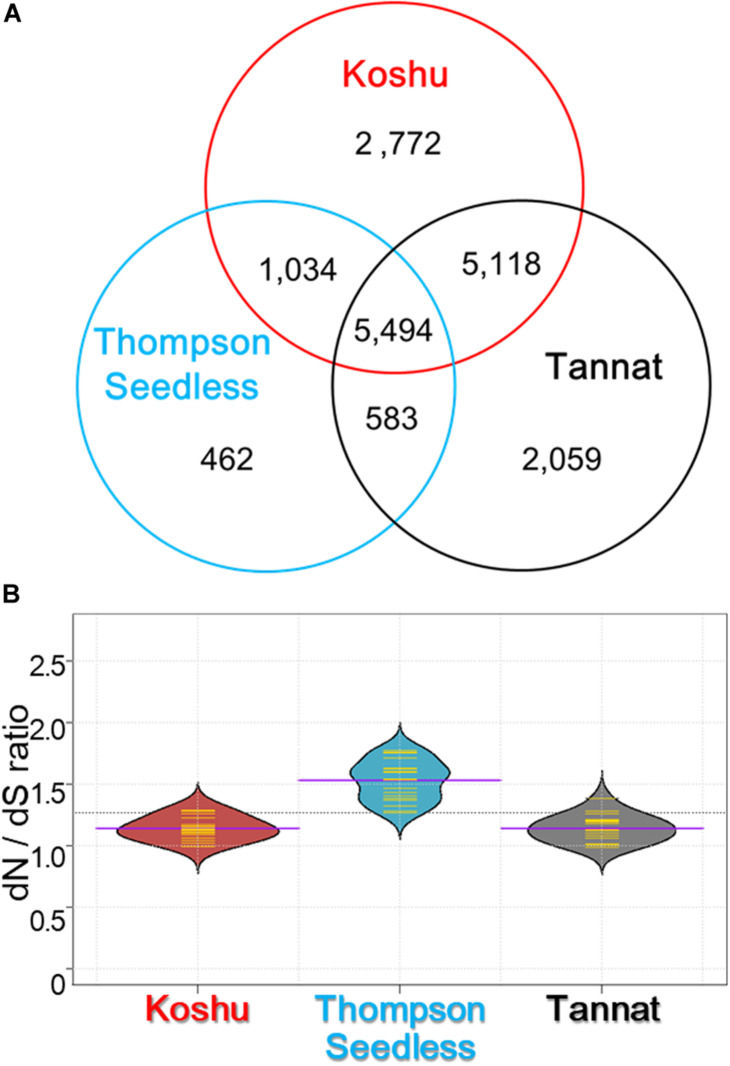
Characterization by gene ontology and KEGG ontology enrichment analyses of non-synonymous variants in Koshu, Thompson Seedless, and Tannat cultivars. **(A)** Venn diagram of the number of genes with non-synonymous variations for the three cultivars. **(B)** Violin plot showing the ratio of non-synonymous substitution to synonymous substitution (dN/dS). The yellow lines indicate the frequency distribution of dN/dS in each chromosome; the purple lines indicate the average dN/dS ratio for the chromosomes; the dashed line indicates the average dN/dS ratio for the three cultivars.

Non-synonymous substitutions were surveyed by detecting amino acid changes in the coding regions using 1,769,160 small variations for Koshu, 1,502,329 for Thompson Seedless, and 2,471,755 for Tannat (Supplementary Table S3). It was found that 5,494 genes with one or more non-synonymous substitutions were common among the three cultivars (Figure 2A). Conversely, 2,772, 462, and 2,059 genes had specific non-synonymous substitutions in Koshu, Thompson Seedless, and Tannat, respectively. Overall, the dN/dS ratio was highest for Thompson Seedless, although the total number of non-synonymous and synonymous substitutions was lowest in this cultivar (Figure 2B and Supplementary Table S4). The dN/dS ratios for Koshu and Tannat were similar. The enriched GO terms from these genes were then categorized by biological process (BP), cellular component (CC), and molecular function (MF).

**FIGURE 3:**
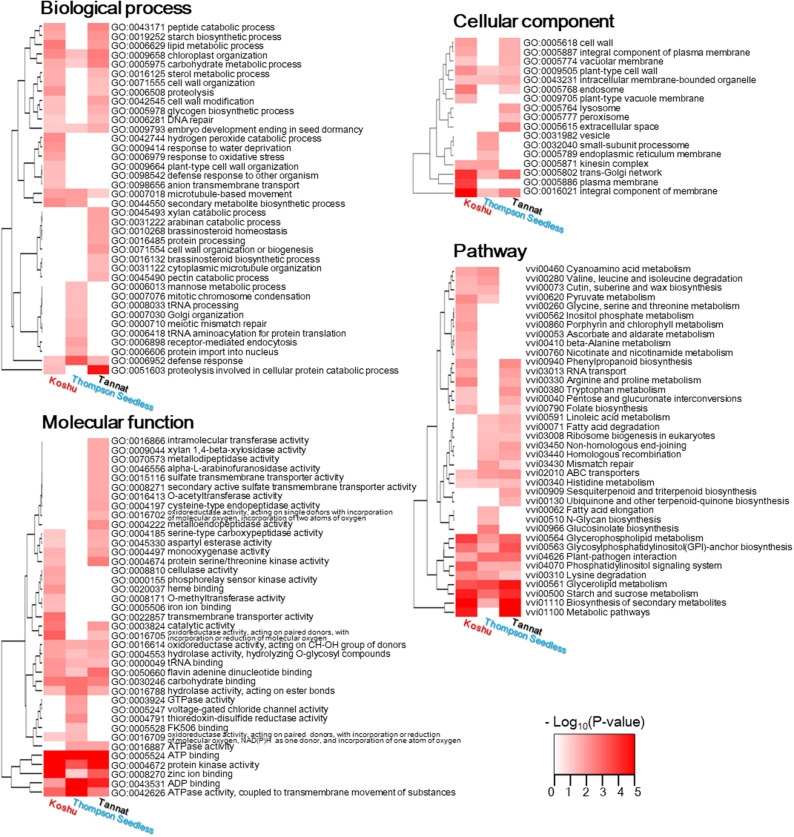
Enrichment of gene sets with non-synonymous variations for the three cultivars. Significantly enriched terms are shown by color gradient. The right side of each heatmap lists profiled significant gene ontology and KEGG ontology terms.

The GO and KO terms that met the significance level (*p* < 0.01) in any one of the three cultivars were displayed on a heatmap (Figure 3 and Supplementary Tables S5, S6). The enrichment identified GO terms with 38 BPs, 17 CCs, 39 MFs, and 38 KO terms. Terms that were enriched in only Koshu included six BPs (“hydrogen peroxide catabolic process,” “response to water deprivation,” “response to oxidative stress,” “plant-type cell wall organization,” “defense response to other organism,” and “anion transmembrane transport”); two CCs (“plant-type vacuole membrane” and “plasma membrane”), six MFs (“cellulase activity,” “phosphorelay sensor kinase activity,” “heme binding,” “O-methyltransferase activity,” “iron ion binding,” and “transmembrane transporter activity”); and six KOs (“glycine, serine and threonine metabolism,” “inositol phosphate metabolism,” “porphyrin and chlorophyll metabolism,” “ascorbate and aldarate metabolism,” “beta-alanine metabolism,” and “nicotinate and nicotinamide metabolism”).

**FIGURE 4:**
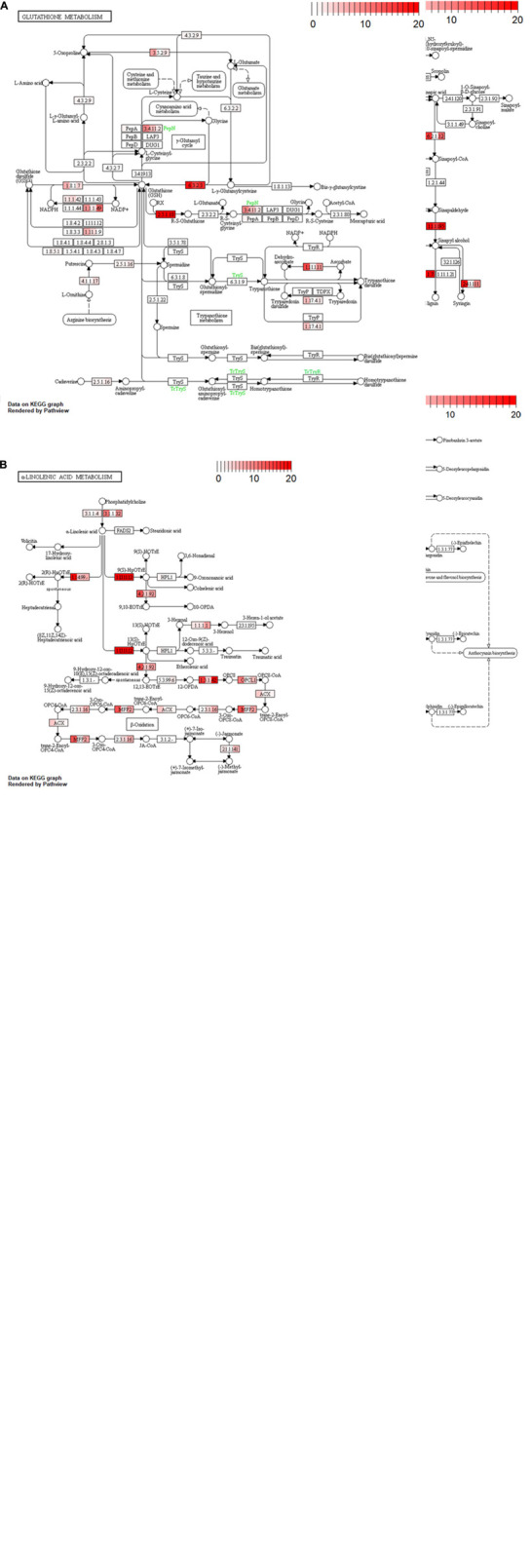
Variant accumulation by non-synonymous substitution in four pathway maps. **(A)** Phenylpropanoid biosynthesis (vvi00940), **(B)** flavonoid biosynthesis (vvi00941), **(C)** glutathione metabolism pathway (vvi00480), and **(D)** α-linolenic acid metabolism (vvi00592). Color in the rectangular boxes indicates variant accumulation levels for Koshu (left), Thompson Seedless (center), and Tannat (right). The numerical value bar at the upper right shows variant burden.

Non-synonymous substitution levels for metabolic genes in the three cultivars were shown by gradient color in each reaction module on a KEGG pathway map (Figure 4). We focused on “phenylpropanoid biosynthesis (vvi00940),” “flavonoid biosynthesis (vvi00941),” “glutathione metabolism (vvi00480),” and “α-linolenic acid metabolism (vvi00592)” as being related to the astringency and/or flavor of wine. Most of the reaction modules for Koshu and Tannat exhibited similar accumulation levels. In comparison, higher variant frequencies in Koshu or Koshu-specific variants were indicated by the reaction modules coding for “4-coumarate-CoA ligase (EC:6.2.1.12),” “coniferyl-alcohol glucosyltransferase (EC:2.4.1.111),” “flavonol synthase (EC:1.14.20.6),” “flavanone 7-O-glucoside 2″-O-beta-L-rhamnosyltransferase (EC:2.4.1.236),” “leucoanthocyanidin reductase (EC:1.17.1.3),” “anthocyanidin synthase (EC:1.14.20.4),” “5-oxoprolinase (ATP-hydrolyzing) (EC:3.5.2.9),” “glutathione reductase (NADPH) (EC:1.8.1.7),” “glutathione peroxidase (EC:1.11.1.9),” “aminopeptidase N (EC:3.4.11.2),” “L-ascorbate peroxidase (EC:1.11.1.11),” “ribonucleoside-diphosphate reductase subunit M1 (EC:1.17.4.1),” “alpha-dioxygenase (EC:1.14.99.-),” “hydroperoxide dehydratase (EC:4.2.1.92),” “acyl-CoA oxidase (ACX),” and “enoyl-CoA hydratase/3-hydroxyacyl-CoA dehydrogenase (MFP2).”

### Structural Variations Based on CNV and PAV

**FIGURE 5:**
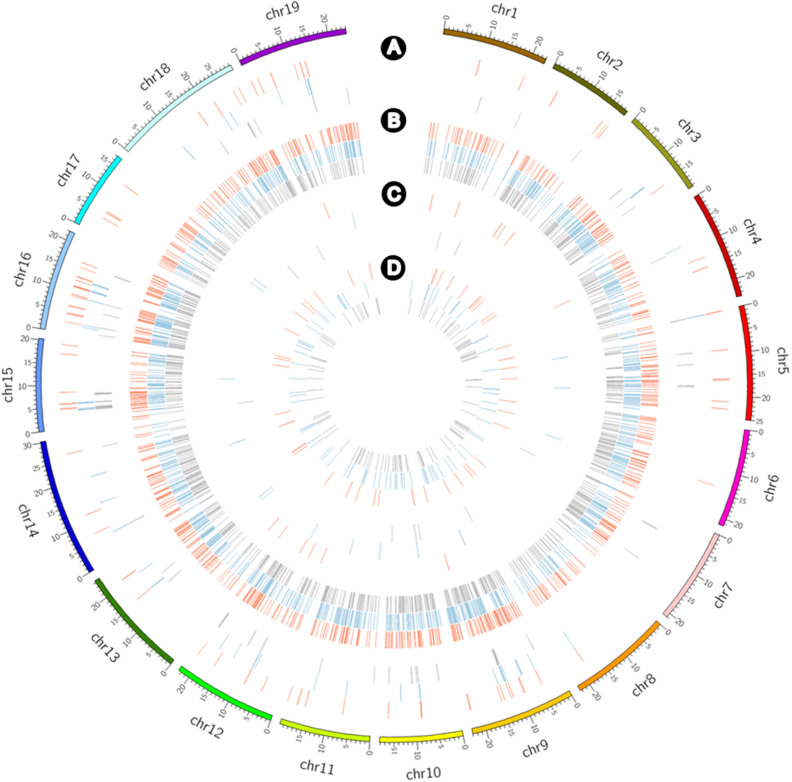
Circos plot showing the detection frequency of CNVs and PAVs for Koshu (red), Thompson Seedless (blue), and Tannat (gray) using Circos (Krzywinski et al., 2009). Distribution of **(A)** CNV duplications, **(B)** CNV deletions, **(C)** presence variations, and **(D)** absence variations.

CNV and PAV analyses to detect large-scale variants revealed deletion and absence variations for the three cultivars (Figure 5). CNVs of ≥4,000 bp obtained by filtering the normalized read depths and *e*-values included 76 duplications with a total of 3,007,000 bp including 135 genes along with 731 deletions with a total of 16,890,000 bp including 331 genes for Koshu; 56 duplications with a total of 2,335,000 bp, including 81 genes along with 899 deletions with a total of 27,391,000 bp including 612 genes for Thompson Seedless; and 66 duplications with a total of 2,673,000 bp including 76 genes along with 831 deletions, with a total of 23,279,000 bp including 569 genes for Tannat (Supplementary Table S7). Alternatively, PAV analysis highlighted 14 presences with a total of 246,550 bp including 5 genes along with 50 absences with a total of 577,303 bp including 23 genes for Koshu; 15 presences with a total of 471,142 bp including 21 genes along with 85 absences with a total of 913,651 bp including 20 genes for Thompson Seedless; and 10 presences with a total of 171,798 bp including 7 genes along with 106 absences with a total of 649,842 bp including 16 genes for Tannat (Supplementary Table S8). Therefore, among the three cultivars, the structural variation of Koshu contained the largest amount of CNV duplication and the smallest amounts of CNV deletion and absence variations.

Enriched GO and KO terms from genes in these structural variant regions were also detected in the three cultivars (Supplementary Tables S9, S10). The number of terms that were enriched in Koshu was less than those in Thompson Seedless and Tannat. The terms that were enriched in Koshu comprised two BPs (“hydrogen sulfide biosynthetic process” and “developmental process involved in reproduction”), one CC (“plasma membrane”), two MFs (“ADP binding” and “adenylylsulfate kinase activity”), and one KO (“plant-pathogen interaction”).

## Discussion

### Koshu Whole Genome Characterization

Comparative genome analysis by resequencing can enhance the understanding of genetic variants and has provided important information pertaining to more than 500 grapevines (Zhou et al., 2017, 2019). Here, we revealed that Koshu appeared to have a different biological background from that of other grape cultivars as determined through phylogenetic analysis of the whole chloroplast genome (Supplementary Figure S2). Next, we performed comparative genome analysis using the cultivars Thompson Seedless and Tannat, the data for which were made available shortly after the publication of the PN40024 reference genome. Small variant data were validated by comparison with high-quality variant information of approximately 1.19 million SNVs for Thompson Seedless and approximately 2.09 million SNVs for Tannat (Da Silva et al., 2013; Di Genova et al., 2014). Whereas the approximately 1.44 million SNVs of Koshu were intermediate between the numbers for the other two cultivars, the total InDel amounts were almost identical among the three cultivars, exhibiting a staircase pattern in the order of decreasing size except for Koshu (Supplementary Figure S3). In addition, the overall heterozygosity level of Koshu was the highest among the three cultivars with chromosome 7 of Koshu in particular exhibiting high heterozygosity (Figure 1 and Supplementary Table S3). Conversely, structural variant data revealed that the total small variant composition of approximately 14.2 Mb for Koshu was less than that of the other two cultivars, although CNV by duplication was the highest in this cultivar (Supplementary Figure 5 and Supplementary Tables S7, S8). Moreover, the SNV, InDel, CNV, and PAV frequencies detected on each chromosome were disproportionate among the three cultivars. Therefore, as with other cultivars, Koshu may also have undergone distinct genomic diversification historically. In this regard, several microsatellite markers have been proposed for identifying individual wine grape cultivars (Cipriani et al., 2008; Galbács et al., 2009). Although chromosome 7 of Koshu has not been reported in such genetic research, the development of microsatellite markers derived therefrom may be effective owing to its high heterozygosity.

### Prediction of Functional Genes

Non-synonymous substitutions and structural variations are considered to affect the *in vivo* functions of Koshu. Among these, we focused on the following variant information related to growth and secondary components.

#### Pathogen and Environmental Stress Responses

Compared to Thompson Seedless and Tannat, the KO term of “plant-pathogen interaction (vvi04626)” was enriched in Koshu (Figure 5 and Supplementary Tables S7, S10). Deletions were found in genes encoding proteins involved in cell death associated with hypersensitive response (HR). HR constitutes a natural immune response of plants to pathogens. A cultivar that shows strong HR tends to exhibit resistance to downy mildew, rather than other pathogens (Staudt and Kassemeyer, 1995; Gindro et al., 2003). Compared with European wine grapes (*Vitis vinifera* L.), Koshu, similar to its ancestor *V. davidii*, is relatively resistant to anthracnose, powdery mildew, and ripe rot. However, Koshu does not readily undergo HR and is more susceptible to downy mildew than *V. vinifera* (Yamakawa, 1983; Wan et al., 2008; Kono et al., 2013, 2018). Hence, these genetic variations may reduce hypersensitive cell death in Koshu.

GO analysis of genes with non-synonymous substitutions in Koshu showed enrichment in “response to water deprivation,” “response to oxidative stress,” and “plant-type cell wall organization” in BP (Figure 3). The protein aspartic protease in guard cell 1 in “response to water deprivation” is involved in conferring drought avoidance in *Arabidopsis thaliana* (Yao et al., 2012). Many peroxidase genes in “response to oxidative stress” affect cell elongation (Liszkay et al., 2004; Vatulescu et al., 2004), stress response (Llorente et al., 2002; Kumar et al., 2012), pathogen resistance (Wally and Punja, 2010), and cell wall strengthening by lignification (Li et al., 2003) and suberization (Bernards et al., 1999). Multiple expansin genes related to “plant-type cell wall organization” promote cell expansion (Schlosser et al., 2008; Suzuki et al., 2014). These results suggested the involvement of these gene variations of Koshu in growth and environmental stress adaptation.

#### Polyphenol-Related Substances

Polyphenols affect the astringency and aroma of wine. Tannat contains six times as much polyphenol as Pinot Noir (Da Silva et al., 2013). Notably, Koshu tends to accumulate similar variations in two biosynthesis pathways (phenylpropanoid: vvi00940 and flavonoid: vvi00941) as Tannat, a red wine cultivar, rather than the Thompson Seedless, a white wine cultivar (Figures 3, 4A,B). We suggest that the slight astringency in Koshu wine is due to the higher total phenolic content of the Koshu berry than of those of European wine grape cultivars (Kobayashi et al., 2011). The Koshu grapevine contains more flavonoids with properties such as antioxidant and antibacterial activity in the stems and leaves than in the skin (Hisamoto et al., 2011). The variant accumulation levels of each gene in the flavonoid pathway (Figure 4B) are consistent with the high expression levels of each gene in a previous RNA-Seq analysis of Koshu shoots (Enoki et al., 2018). These features of Koshu, which are similar to those of a red wine cultivar, might be influenced by variant accumulation in polyphenol-related pathways as a result of the non-synonymous variations in the Koshu genome.

#### Flavor Components

3-Mercaptohexanol (3MH), which was first identified in the cultivar *V. vinifera* cv. Sauvignon blanc that exhibits grapefruit and passion fruit aroma (Tominaga et al., 1998), is also present in Koshu wine and is considered one of the positive features of Koshu. Previous reports of 3MH metabolism have revealed that a 3MH precursor (3MH-S-glut) is generated by the glutathione-S-transferase-catalyzed synthesis of glutathione and hexenal during Koshu berry ripening (Kobayashi et al., 2011). The amount of accumulated variations in each gene of the “glutathione metabolism (vvi00480)” pathway is almost consistent with the high expression of each gene in our previous report (Figure 4C; Enoki et al., 2018). In fact, total glutathione is present in Koshu in relatively large amounts (Okuda and Yokotsuka, 1999). Thus, these variants are considered to contribute to the increase in glutathione synthesis in Koshu. Hexenal is a C6 compound produced by the C18 metabolic pathways. Hexenal contents are smaller in Koshu than in the other white wine cultivars (Arita et al., 2017; Bahena-Garrido et al., 2019). Moreover, in the substrate branch of 13(S)-HpOTrE in the C18:3 metabolic pathway (vvi00592) (Figure 4D), differences in the accumulation of variations in many genes encoding allene oxide synthase (EC:4.2.1.92), but not hydroperoxide lyase (EC:4.1.2.-), were detected only in Koshu (Figure 3 and Supplementary Table S5). Hydroperoxide lyase generates hexenal, and allene oxide synthase acts on the jasmonic acid biosynthesis pathway (Kessler et al., 2004; Chehab et al., 2008). Hence, Koshu tends to produce jasmonic acid in this branching pathway over hexenal, and 3MH production in Koshu may be dependent on the amount of hexenal rather than glutathione.

## Conclusion

Koshu showed a phylogenetically distinct relationship compared to that of other European grapes and exhibited genomic variations and structures that differed from the two comparative grape cultivars, Thompson Seedless and Tannat. In particular, evidence existed of high heterozygosity for chromosome 7. The high heterozygosity may be because *V*. *davidii*, one of the parents of Koshu, may harbor resistance genes. Our findings also supported our previous transcriptional report (Enoki et al., 2018) investigating whether Koshu enhances the expression of defense genes the phenylpropanoid pathway. Similarly, the polyphenol genes differentially enriched in Koshu might suggest a higher phenolic content in the berries and wine. Although wine taste is affected by terroir, wine grape genomes clearly affect wine quality and play a vital role in viticulture. By further expanding and improving the genomic information of wine grapes, the improvement of wine cultivars can be expected through application of genome-wide association studies, genomic selection, and marker-associated selection.

## Data Availability Statement

The datasets generated for this study can be found in the NCBI SRA database (accession number SRR11234330).

## Author Contributions

KT and SE wrote the manuscript. KT and YH designed and performed genomic data analysis. SE and SS prepared the materials and data and offered advice regarding the study. SS planned and supervised the study. All authors have read and approved the final manuscript.

## Conflict of Interest

The authors declare that the research was conducted in the absence of any commercial or financial relationships that could be construed as a potential conflict of interest.

## Footnotes

1 http://www.vivc.de

2 https://www.ncbi.nlm.nih.gov/sra/

3 https://www.ncbi.nlm.nih.gov/refseq/

4 https://www.ncbi.nlm.nih.gov/genome/

5 https://david.ncifcrf.gov

6 http://shiny.chemgrid.org/boxplotr/
