# Supplementary figures and images for: Genomic Characterization of the Japanese Indigenous Wine Grape Vitis sp. cv. Koshu
Source: Front Plant Sci. 2020 Nov 5;11:532211. doi: 10.3389/fpls.2020.532211 (PMC7720679; doi:10.3389/fpls.2020.532211)

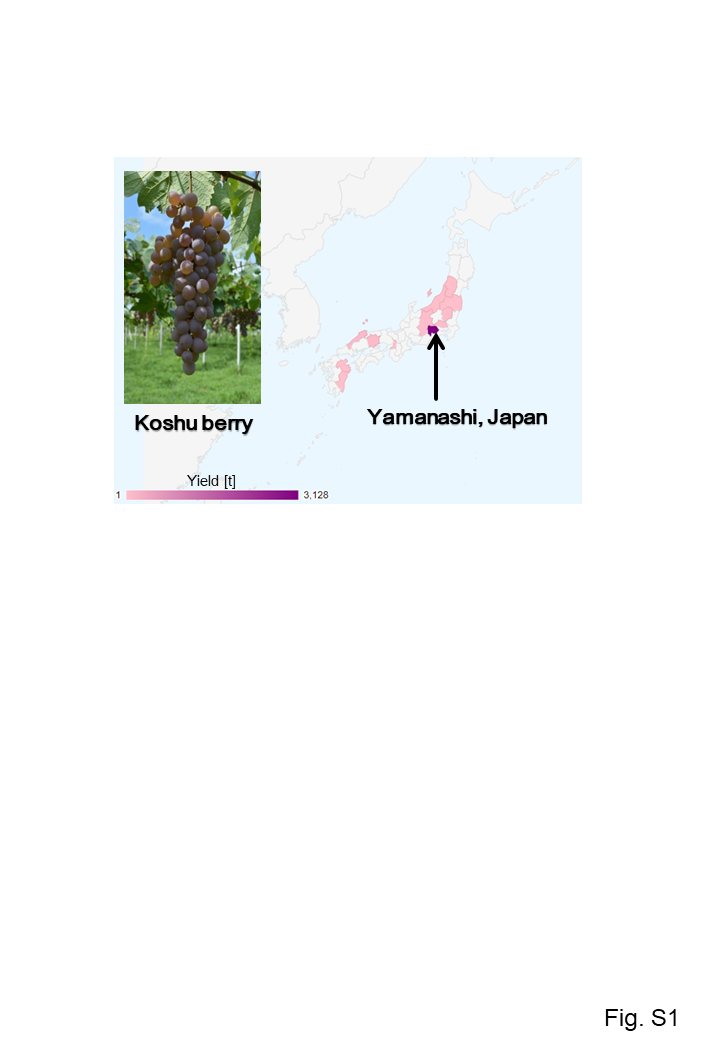

Supplement: Supplementary file 2 [file Image_1.TIF]

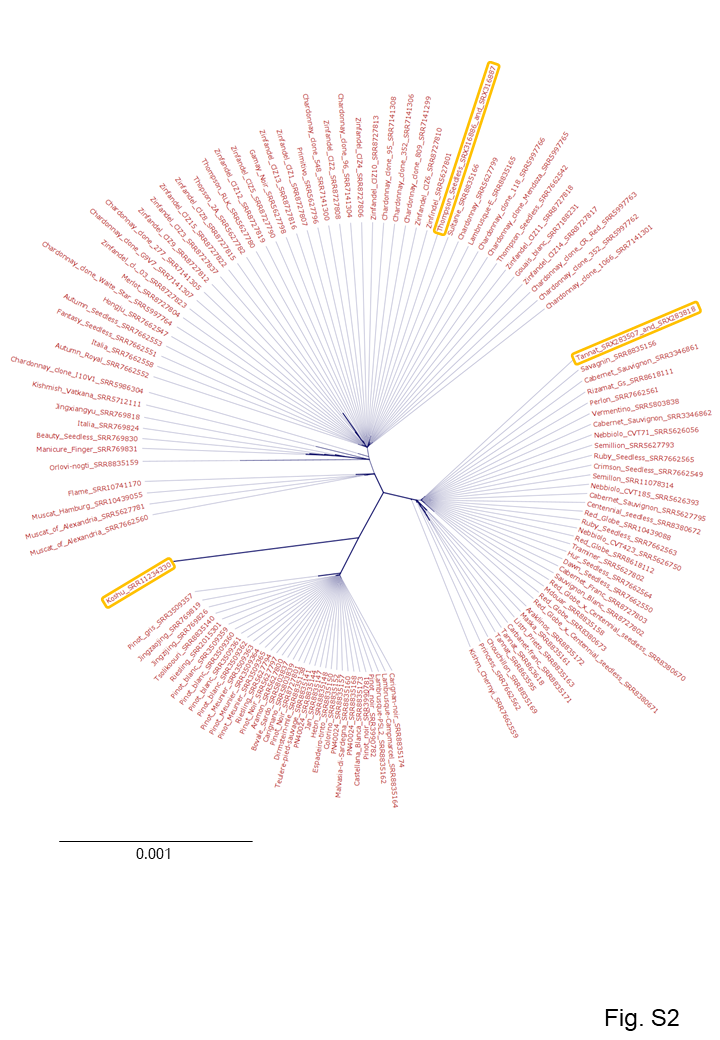

Supplement: Supplementary file 3 [file Image_2.TIF]

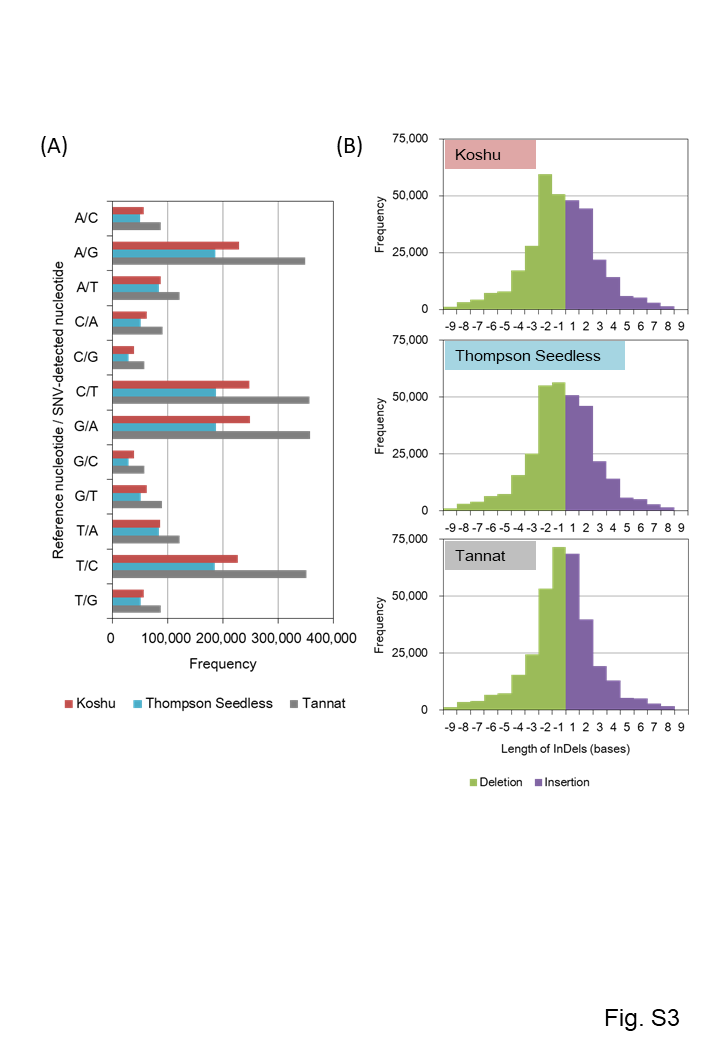

Supplement: Supplementary file 4 [file Image_3.TIF]
